# The presence of persistent synovial inflammation after “Eradication” unmasks the “Unseen” dormant state of infection allowing the prediction of infection free survival in total joint replacements

**DOI:** 10.1186/s12967-025-07425-y

**Published:** 2025-12-12

**Authors:** Robert Manasherob, Shay I. Warren, Christopher M. Flanagan, Prerna Arora, Lyong Heo, Christopher J. Moore, Simon K. H. Chow, Z. Ngalo Otieno-Ayayo, Daisuke Furukawa, William J. Maloney, David W. Lowenberg, Stuart B. Goodman, Derek F. Amanatullah

**Affiliations:** 1 Department of Orthopaedic Surgery, Stanford University, 450 Broadway Street, Redwood City, CA 94025 USA; 2 Stanford School of Medicine, Department of Orthopaedic Surgery, Biomedical Innovations Building 240 Pasteur Drive, Palo Alto, CA 94304 USA; 3 Genetics and Bioinformatics Service Center, Stanford University, 3165 Porter Drive, Palo Alto, CA 94304 USA; 4 Stanford School of Medicine, Department of Genetics, Cooper Lane, Alway Building Palo Alto, Stanford, CA 94304 USA; 5 School of Science, Agriculture, and Environmental Studies, Rongo University, P.O. Box 103, Rongo, 40404 Kenya; 6 Department of Medicine, Division of Infectious Diseases and Geographic Medicine, Stanford University, 300 Pasteur Drive, Lane Building, Redwood City, CA 94025 USA

**Keywords:** Dormant infection, Proteomics, Biofilm, Small colony variants, Persister cell, Arthroplasty, Joint replacement, Periprosthetic joint infection, Musculoskeletal infection, Implant-associated infection

## Abstract

**Introduction:**

The conventional clinical criteria for diagnosing a periprosthetic joint infection (PJI) rely on acute inflammatory readouts such as erythrocyte sedimentation rate (ESR), C-reactive protein (CRP), and synovial white blood cell counts. These metrics only detect actively septic joint replacements and may miss detecting biofilm-embedded bacteria that suppress neutrophil signaling and persist as a “hidden” subset of implants with a dormant infection. We hypothesize that previously infected joint replacements have a high prevalence of dormant infection that can be distinguished from aseptic revision joint replacements (replaced for instability, loosening, wear, and fracture) by the persistent inflammatory response within synovial fluid and/or circulating plasma, and that detecting a dormant infection indicates an increased risk of infection relapse.

**Methods:**

This is an observational cohort study using synovial fluid and plasma proteomics of 96 immuno-oncology mediators (Olink Proteomics, Sweden) with three-year clinical follow-up from a single academic medical center (Stanford University, USA). Thirty patients undergoing revision joint replacement: culture-positive actively septic joint replacements (*n* = 7), aseptic revision joint replacements (*n* = 12), and re-implantations of joint replacements previously classified as infection-free by 2018 Musculoskeletal Infection Society (MSIS) criteria (*n* = 11). Differential expression, unsupervised clustering, Euclidean distance mapping, principal-component analysis, and gene-set variation analysis were used to define the inflammatory signature of dormant infection present in joint replacements with a prior infection. The identified biomarkers of dormant infection were correlated with the three-year incidence of infection relapse.

**Results:**

Eight of eleven MSIS-cleared joint replacements (73%) clustered with culture-positive active infections despite normal ESR, CRP, and scant synovial neutrophils revealing the synovial inflammatory signature of dormant infection. A nine-analyte synovial panel consisting of PDGF-B, CXCL5, CXCL11, MCP-2 (CCL8), ANGPT1, TIE2, EGF, NOS3, and Gal-1 distinguished dormant infection from truly aseptic cases with 100% specificity and positive predictive value (sensitivity 22%, negative predictive value 74%). Synovial CXCL5 over-expression was a universal hallmark of both active and dormant infection, whereas matched plasma profiles showed no discriminatory power for all immuno-oncology mediators tested. Dormant infections exhibited downregulation of granulocyte activation and T-cell proliferation pathways (FDR < 0.001), mirroring immune evasion programs seen in cancer microenvironments. After a mean of 3 years follow-up, infection relapse occurred in 22% of the biomarker-positive dormant infections, but relapse did not occur in any of the biomarker-negative aseptic cases.

**Discussion:**

Profiling of the persistent inflammatory response within the synovial fluid of two-stage re-implantations classified as “infection-free” by the MSIS criteria unmasked a clinically silent reservoir of biofilm-embedded bacteria that suppress clinical diagnostic criteria of active infection and define the novel clinical state of dormant infection. We used that profile to identify a novel culture-free panel of rule-out biomarkers for determining which re-implantations were safe from infection relapse. These findings challenge the use of conventional clinical diagnostic criteria, which are over-reliant on acute phase reactants and neutrophil recruitment, and creates a new prognostic clinical paradigm that now includes a future with precision, immune-guided management of dormant infections likely present in many implant-associated infections.

**Supplementary Information:**

The online version contains supplementary material available at 10.1186/s12967-025-07425-y.

## Introduction

Bacteria form biofilms on orthopaedic implants (e.g., plates, screws, joint replacements), non-orthopaedic implants (e.g., pacemakers, abdominal mesh, breast implants, catheters), and biologic tissues that act as “implants” (e.g., endocarditis, osteomyelitis, cellulitis, sinusitis, chronic wounds) [1–5]. The annual healthcare cost attributed to biofilm exceeds $386 billion globally and biofilm accounts for 80% of microbial infections, nearly all of which end up requiring surgical treament [1, 6, 7]. Biofilm is composed of bacterial communities encased in a protective extracellular matrix. Biofilm-resident bacteria (e.g., small colony variants, sessile cells, and/or persister cells) are notoriously difficult to culture, remarkably tolerant to antibiotics, and capable of evading phagocytosis [8–11]. When phagocytized, biofilm-resident bacteria dramatically alter cytokine production, modify macrophage polarization, compromise antigen presentation, and inhibit neutrophil function [9, 12–17]. 

The ability of biofilm-resident bacteria to regulate the immune response, coupled with the overreliance of current diagnostic criteria for infected joint replacements on the local and systemic neutrophil response, means that biofilm-resident bacteria can establish a dormant infection that may be misclassified as uninfected [12, 18]. Next-generation sequencing of fluid from antibiotic-laden spaces from joint replacements with a prior infection show the continued presence of biofilm-resident bacteria (indicative of a dormant infection) that are next to impossible to culture and thus may be misclassified as uninfected [18–21]. The high infection relapse rate after a revision joint replacement with a prior infection is consistent with the hypothesis that patients may harbor dormant infections that emerge into acute infections after subsequent surgery [21]. Given the surgical etiology, access to synovial samples, and staged spacer-related treatment, revision joint replacements with a prior infection are the ideal population to detect implant-associated dormant infection [22]. 

The accurate identification of dormant infection is of critical importance for cost-effective management of implant-associated infection. The inability to effectively diagnose a dormant infection relegates most clinical work on infection to the diagnosis of only the most advanced disease state - systemic sepsis and actively infected joints [23]. The inability to effectively diagnose a pre-disease state, like dormant infection, prevents the prediction of future active infections that currently appear to emerge sporadically. The sporadic presentation may be due to the emergence of active inflammatory signals that trigger conventional clinical diagnostic criteria for a periprosthetic joint infection (PJI) that develop from a more dormant inflammatory state (i.e., conversion of the pre-disease state to the advanced disease state) [24, 25]. We hypothesize that revision joint replacements with a prior infection have a high prevalence of dormant infections that can be distinguished from uninfected joint replacements by the inflammatory response in synovial fluid and/or circulating plasma and that detection of a dormant infection reveals an increased risk of infection relapse.

This hypothesis builds on our previous work using single-cell transcriptomics to reveal the cellular signature and gene expression characteristics of dormant infection. The cellular signature of dormant infection has many hallmarks of active infection, including a reduction in both classically activated M1 and alternatively activated M2 macrophages coupled with an increase in classical monocytes, myeloid and plasmacytoid dendritic cells, natural killer cells, and regulatory T-cells, but lacked the systemic or local neutrophil recruitment commonly seen in active infections [12]. The characteristic gene expression of dormant infection suggested the activation of interleukin (IL)-17 and tumor necrosis factor (TNF) pathways that drive synovial CXCL5 expression, which should stimulate neutrophil recruitment. However, dormant infections also downregulated neutrophil extracellular trap (NET) formation as well as immune checkpoint regulation, classically activated M1 macrophage polarization, and T-cell response [12]. To remain unbiased for the purpose of this work, we defined a dormant infection as a joint replacement with a prior infection as diagnosed by the 2018 Musculoskeletal Infection Society (MSIS) criteria that underwent implant resection and completion of 6 weeks of microbe-specific intravenous antibiotic therapy and were subsequently reimplanted after being deemed “infection free” using the same 2018 MSIS criteria at the end of a more than 6 week antibiotic holiday, and at reimplantation had a synovial inflammatory response that mimicked an active infection as determined by unsupervised clustering of the synovial proteome [26, 27]. 

## Methods

### Patient samples

**Fig. 1:**
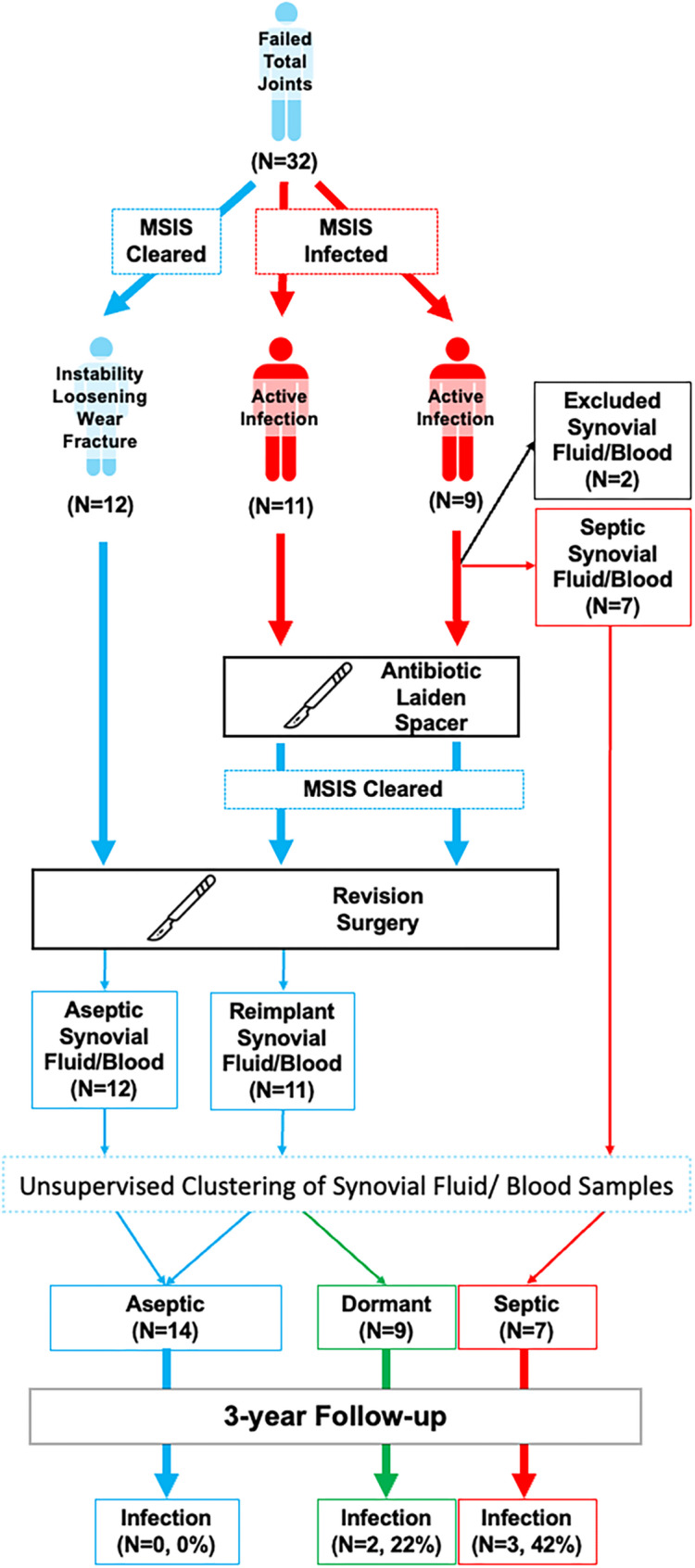
Clinical treatment pathway as determined by the current diagnostic criteria. Synovial protein and blood samples were drawn in Musculoskeletal Infection Society (MSIS) infected cases (septic, *n*=9), cases of instability, loosening, wear, fracture (MSIS-cleared, aseptic, *n*=12), and Musculoskeletal Infection Society (MSIS)-cleared previously septic cases (*n*=11) for proteomic analysis of blood and synovial fluid then followed for a minimum of 3 years to evaluate the rate of infection relapse after unsupervised clustering of the immune-oncology proteomic analytes

After institutional review board approval at Stanford University (Biosafety #4328, Clinical Safety #54462 and #72855), we collected matched whole blood and aspirated synovial fluid in separate purple top tubes from 32 patients. We excluded one patient with a quality control warning in the synovial fluid sample and another patient as a potential outlier based on the interquartile range of synovial fluid sample medians. The remaining 30 paired patient samples (94%, Table 1; Fig. 1) comprised 14 (47%) total hip and 16 (53%) total knee replacements. Seven joint replacements had an active infection with *Staphylococcus aureus*,* S. lugdunesis*,* S. epidermidis*,* Escherichia coli*,* or Proteus mirabilis* (23% septic revisions, follow-up: 2.6 ± 1.1 years after resection and retention of a durable 1.5 stage antibiotic-laden spacer), 12 joint replacements without an infection (39% aseptic revisions for instability, loosening, wear, or fracture, follow-up: 3.2 ± 0.3 years after aseptic revision surgery, *p* = 0.200, t-test v. infections), and 11 joint replacements with a prior *S. aureus*, *S. lugdunesis*, S. *epidermidis*, or *Salmonella enterica* infection deemed “infection-free” after spacer placement by the 2018 MSIS criteria after completion of >6 weeks of oral or intravenous antibiotics and a >6 week antibiotic free holiday (37% MSIS-cleared, follow-up of 3.0 ± 0.2 years after definitive reimplantation, *p* = 0.170, t-test v. infections, Fig. 1) [26]. Continuous demographic and clinical variables are reported as mean and standard deviation (SD) unless otherwise noted and compared via independent t-tests with the two-sided level of significance set to α < 0.05. Categorical demographic and clinical variables are reported as numerical count and percentages and compared via a chi-square or Fisher’s exact tests with the level of significance set to α < 0.05.

**Table 1:** Demographic and physiologic characterization of joint replacement populations distributed by infection status

	Infection status			
	All (*n* = 30)	Active (*n* = 7)	Prior (*n* = 11)	None (*n* = 12)
**Demographics**				
Age, years (mean ± SD)	62.5 ± 14.4	55.4 ± 15.1	61.0 ± 14.1	68.2 ± 13.6
Knee Replacements, number (%)	14 (46.7)	2 (28.6)	6 (54.5)	7 (58.3)
Hip Replacements, number (%)	16 (53.3)	5 (71.4)	5 (45.5)	5 (41.7)
Side, Right, number (%)	15 (50.0)	4 (57.1)	5 (45.5)	6 (50.0)
ASA Classification, units (mean ± SD)	2.6 ± 0.5	2.7 ± 0.5	2.6 ± 0.6	2.5 ± 0.5
Body Mass Index, kg/m^2^ (mean ± SD)	31.0 ± 8.6	30.9 ± 13.4	31.5 ± 6.8	27.4 ± 5.5
Smokers, active, number (%)	5 (16.7)	2 (28.6)	2 (18.2)	1 (8.3)
**Medical comorbidities**				
Anxiety/Depression, number (%)	8 (26.7)	2 (28.6)	3 (27.3)	3 (25.0)
Abuse Disorder, number (%)	5 (16.7)	2 (28.6)	1 (9.1)	2 (16.7)
Diabetes, number (%)	8 (26.7)	3 (42.9)	4 (36.4)	1 (8.3)
Hypertension, number (%)	18 (60.0)	5 (71.4)	7 (63.6)	6 (50.0)
Hyperlipidemia, number (%)	4 (13.3)	2 (28.6)	2 (18.2)	0 (0.0)
Coronary Artery Disease, number (%)	3 (10.0)	1 (14.3)	0 (0.0)	2 (16.7)
Arrhythmia, number (%)	4 (13.3)	1 (14.3)	1 (9.1)	2 (16.7)
Congestive Heart Failure, number (%)	1 (3.3)	0 (0.0)	1 (9.1)	0 (0.0)
Pulmonary Disease, number (%)	3 (10.0)	1 (14.3)	2 (18.2)	0 (0.0)
Obstructive Sleep Apnea, number (%)	7 (23.3)	1 (14.3)	5 (45.5)	1 (8.3)
Cerebral Vascular Accident, number (%)	1 (3.3)	0 (0.0)	0 (0.0)	1 (8.3)
Anemia, number (%)	1 (3.3)	0 (0.0)	0 (0.0)	1 (8.3)
Osteoporosis, number (%)	1 (3.3)	0 (0.0)	1 (9.1)	0 (0.0)
Hypothyroidism, number (%)	3 (10.0)	0 (0.0)	1 (9.1)	2 (16.7)
Prior Thrombosis or Embolism, number (%)	7 (23.3)	3 (42.9)	3 (27.3)	0 (0.0)
Hepatitis, number (%)	2 (6.7)	0 (0.0)	1 (9.1)	1 (8.3)
Renal Failure, number (%)	1 (3.3)	0 (0.0)	1 (9.1)	0 (0.0)
Cancer, number (%)	1 (3.3)	0 (0.0)	0 (0.0)	1 (8.3)

ASA: American Society of Anesthesiologists, SD: Standard Deviation, *p* < 0.05 denoted as ^*^ for t-test active to prior, ^†^ for t-test prior to none, ^‡^ for active to none, or ^#^ for Chi-square

### Proximity extension assay

The expression of 96 plasma proteomic analytes was determined using a highly multiplexed platform (Immuno-Oncology Panel v.3112 Lot#: B12204, intra-control-value 5%, Olink Proteomics, Uppsala, Sweden) from intra-operative synovial fluid and pre-operative plasma samples matched to the tissue samples from each patient via proximity extension [28]. This proximity extension assay (PEA) allows high-throughput, multiplexed protein quantification with high specificity and sensitivity. Normalized protein expression (NPX, log_2_) data were exported from Olink Software and loaded into R (version 4.3.2) using the read_NPX function from the OlinkAnalyze R package (version 4.0.1) [29]. Quality control was performed and excluded one sample with a quality control warning and another as a potential outlier based on the interquartile range of sample medians. Statistical testing for differential protein expression was performed using olink_ttest in the OlinkAnalyze R package [29]. Statistically significant expressions with an adjusted p-value < 0.05 after Welch two-sample t-test were labeled in each of the volcano plots.

### Unsupervised clustering to identify dormant infection

There are three reasons we intentionally utilized unsupervised clustering to create a novel immunologic definition of a dormant infection based on a persistent synovial inflammation after a prior culture-positive implant-associated infection [27]. First, biofilm and bacterial persistence often obscure bacterial culture results, making them a highly unreliable diagnostic tool [8, 30, 31]. Second, advanced sequencing techniques are subject to surgical sampling bias and the detection of contaminating bacterial DNA [32, 33]. Third, our goal was not to define a new test to replace synovial or tissue culture, but to instead define the persistent inflammatory signature required to influence the immunologic definition of infection itself. This is particularly important, because the MSIS, [26, 34] European Bone and Joint Infection Society (EBJIS), [35, 36] Infectious Disease Society of America (IDSA), [37] and International Consensus Meeting (ICM) [38, 39] criteria are essentially immunologic criteria that represent the local and systemic neutrophil response [40].

To identify a persistent inflammatory signature indicative of dormant infections, we utilized Euclidean distance-based clustering and principal component analysis (PCA) [27, 41]. Euclidean distance-based clustering provides a straightforward way to measure the similarity between samples, making it easier to interpret and understand their relationships. Because small changes in features can directly impact the Euclidean distance, it is more sensitive to local features, than nearest-neighbor decisions [42]. Euclidean distance is also scale-sensitive, meaning that features with larger numeric ranges can have a greater impact on the distance calculations, which is important for high-dimensional data, like proteomics. PCA reduces the number of features in a dataset while preserving as much variance as possible. This is particularly useful when dealing with high-dimensional data, where many features may be correlated or redundant. By transforming the original variables into a smaller set of uncorrelated variables, called principal components, PCA simplifies the dataset without losing significant information. By reducing the number of dimensions, PCA allows for easier visualization of complex datasets. It helps in identifying patterns, trends, and outliers in the data, making it easier to interpret and analyze. PCA is effective in situations where multicollinearity exists among features. By transforming correlated variables into uncorrelated principal components, PCA mitigates issues that arise from multicollinearity, which can negatively impact the performance of regression models and other machine learning algorithms. This can help PCA denoise data by filtering out less significant components that may represent noise rather than useful information. This enhances the quality of the data used for analysis and modeling. Clustering was visualized with the heatmap function from the ComplexHeatmap R package (version 2.18.0), and PCA was performed using the prcomp function from the stats R package (version 4.3.2) [43].

### Gene set variation analysis

Gene Set Variation Analysis (GSVA) reduces the noise and dimensionality of the proteomic data providing a more interpretable summary of pathway-level insights. GSVA has the added benefit of being robust, flexible, and offers increased power to detect subtle changes in activity when compared to traditional GSE methods [44]. Gene set scores for Gene Ontology (GO) terms and Kyoto Encyclopedia of Genes and Genomes (KEGG) pathways were computed using the gsva function from the GSVA R package (version 1.50.5) [44]. Differential expression analysis was performed using empirical Bayesian statistics with the eBayes function in the limma R package (version 3.58.1)[45]. Adjustments for multiple comparisons were applied using the Benjamin-Hochberg method to control the False Discovery Rate (FDR).

### Identification of biomarkers of dormant infection

To identify the most informative proteins that distinguish the dormant infection state from the active and no infection states, we used the area under the receiver operating characteristic curve (AUC) and the random forest algorithm. AUC values were computed using the roc and auc functions in the pROC R package (version 1.18.5)[46]. Random Forest modeling was performed using the randomForest function in the randomForest R package (version 4.7.1.2), with the default values of ntree and mtry set to 500 and 30, respectively [47]. These defaults were used to set the structure of the forest and the percentage increase in mean squared error (%IncMSE) was used as the variable-importance metric for each protein. Proteins were then ranked based on AUC and %IncMSE values, and those with the highest scores were selected as the most informative proteins for the accurate detection of a dormant infection.

## Results

### Synovial inflammation persists after infection clearance

**Fig. 2:**
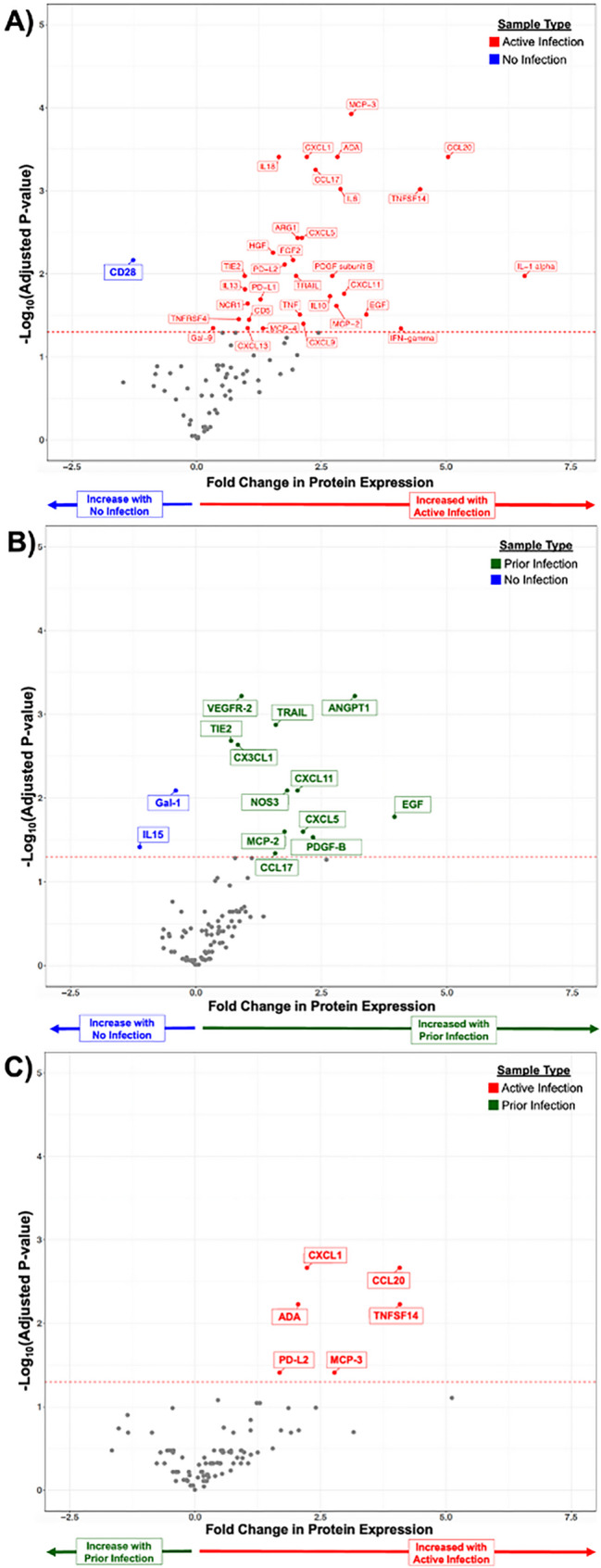
Synovial inflammatory protein expression differs by infected state. Synovial protein expression as detected by proximity extension assay from joint replacements with an (**A**) active infection or (**B**) a prior infection in relationship to no infection as well as in relationship to each other (**C**)

Clinically infected synovial-fluid proteomes segregated sharply from samples that showed no signs of prior infection. As expected, culture-positive joint replacements with an active infection displayed a broad inflammatory signature (Fig. 2A). Strikingly, MSIS-cleared joint replacements undergoing re-implantation showed an inflammatory profile almost indistinguishable from actively infected joint replacements (Fig. 2B, Supplementary Table 1). Only six mediators, ADA, CXCL1, CCL20, MCP-3, PD-L2, and TNFSF14, differed between the “infection-free” and actively infected groups (Fig. 2C). The near-identity of the synovial proteomic fingerprints of the “MSIS-cleared” and septic joint replacements, despite normal systemic acute-phase reactants and minimal synovial neutrophil recruitment (Table 2), suggests that many re-implantations of prior infections may be mislabeled as “infection-free” and still harbor a biologically active, yet clinically “dormant,” infection. Plasma proteomics did not discriminate among the three groups (data not shown), underscoring the peri-implant space as the critical compartment for detecting implant-associated dormant infection as a unique pathologic entity.

**Table 2:** Serum scute phase reactants and synovial neutrophil-based markers of infected joint replacement populations distributed by infection status

Marker of active infection	Infection status		
	Active (*n* = 7)	Prior (*n* = 11)	None (*n* = 12)
Erythrocyte Sedimentation Rate, mm/h (mean ± SD)^*,‡^	71.0 ± 48.1	16.9 ± 11.5	19.0 ± 22.8
C-reactive Protein, mg/dL (mean ± SD)^*,‡^	9.6 ± 8.3	0.4 ± 0.2	0.7 ± 0.9
Synovial White Blood Cell Count, Kcells/mL (mean ± SD)^*,‡^	15.3 ± 12.1	0.5 ± 0.9	0.3 ± 0.1
Percent Polymorphonuclear Neutrophils (mean ± SD)^*,‡^	70.2 ± 5.1	60.7 ± 6.4	55.9 ± 7.5
Culture Positive, number (%)^#^	6 (85.7)	0 (0.0)	0 (0.0)

SD: Standard Deviation, *p* < 0.05 denoted as ^*^ for t-test active to prior, ^†^ for t-test prior to none, ^‡^ for active to none, or ^#^ for Chi-square

### Persistent synovial inflammation unmasks dormant infection

**Fig. 3:**
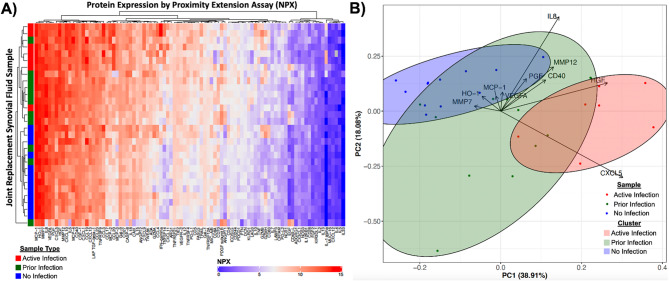
Prior infections display heterogeneity and CXCL5 expression is a hallmark of dormant and active infection. **A**) Hierarchical clustering of synovial fluid samples from joint replacements (left, y-axis) with an active infection (red), a prior infection (green), or no infection (blue) by synovial protein expression as detected by proximity extension assay and represented as a clustered heatmap (top, x-axis). **B**) Principal component analysis showing a lack of distinction between active infection (red), a prior infection (green), and no infection (blue) with CXCL5 expression driving the resolution of active infection seen by Euclidean distance-based clustering

Unsupervised clustering of the synovial proteome stratified the 11 MSIS-cleared re-implantations into 3 molecular subgroups (Fig. 3A). Eight cases (73%) grouped tightly with culture-positive joint replacements with an active infection, despite normal ESR/CRP and scant synovial neutrophils, and are hereafter designated as “dormant infections.” Two cases (18%) clustered with aseptic joint replacements, whereas one sample (9%) lay equidistant from both poles and was excluded from downstream analyses. CXCL5, a neutrophil-attracting chemokine, was similarly elevated in active (12.8 ± 0.7 NPX) and dormant infection samples (12.9 ± 1.1 NPX, *p* = 0.780) when compared to the aseptic joint replacements and the MSIS-cleared samples that clustered with them (10.5 ± 1.6 NPX, *p* < 0.001 versus dormant, *p* = 0.002 versus active). Hence, CXCL5 expression can discriminate the dormant and active states from the uninfected state (Fig. 3B).

**Fig. 4:**
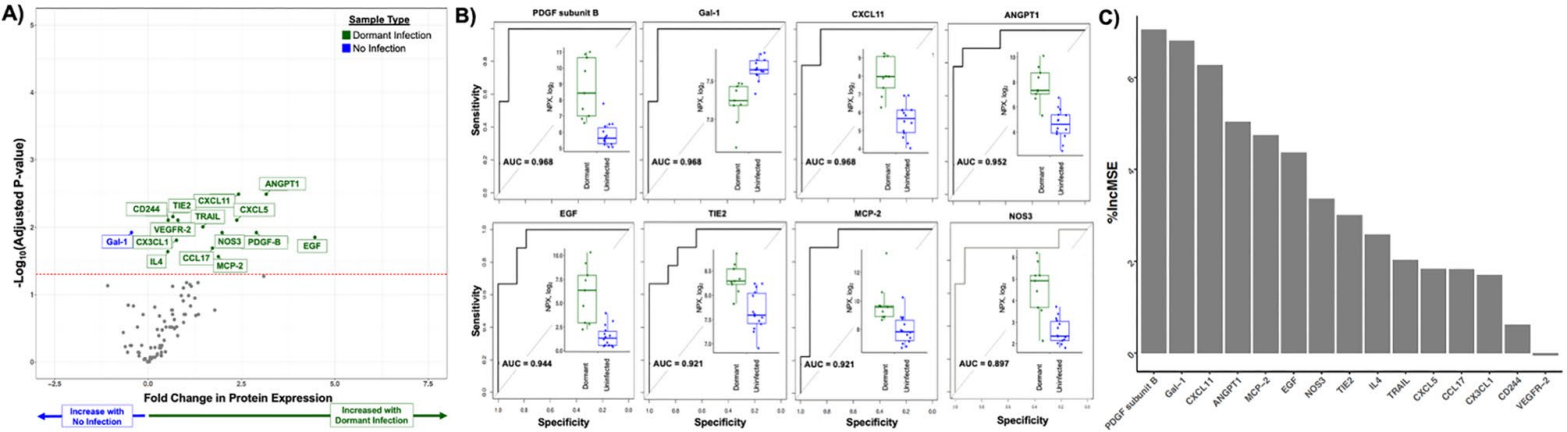
Candidate biomarkers that distinguish dormant infections from uninfected joint replacements. **A**) Synovial inflammatory protein expression as detected by proximity extension assay from joint replacements with a dormant infection in relationship to joint replacements with no infection demonstrating the expression of 15 candidate biomarkers for dormant infection in the synovial fluid of joint replacement patients. **B**) Accuracy as measured by area under the curve (AUC) for all candidate biomarkers with a better accuracy than CXCL5, as identified by Euclidean distance-based clustering, for identifying a dormant infection. Embedded box and whisker plots showing the expression of each candidate biomarkers in normalized protein expression (NPX, log_2_) for patients with a dormant infection (green) and without an infection (blue). **C**) Importance of each candidate biomarker for dormant infection in percentage increase in mean squared error (%IncMSE)

Comparing dormant infections with aseptic joint replacements identified 15 candidate biomarkers that can discriminate the dormant from the uninfected state (Fig. 4A). Random-forest modelling ranked 7 of these, PDGF-B, Gal-1, CXCL11, ANGPT1, EGF, TIE2, and MCP-2 (CCL8), as the most informative with individual accuracies exceeding 90%, each surpassing CXCL5 (AUC = 0.897; %IncMSE = 1.7) for diagnostic power (Fig. 4B-C, Supplementary Table 2). Both CD244 and NOS3 matched CXCL5 in accuracy, with NOS3 showing greater model importance (%IncMSE = 4.5, Fig. 4B-C, Supplementary Table 2). None of the discriminatory proteins was detectably altered in plasma (data not shown), again underscoring the value of local sampling.

**Fig. 5:**
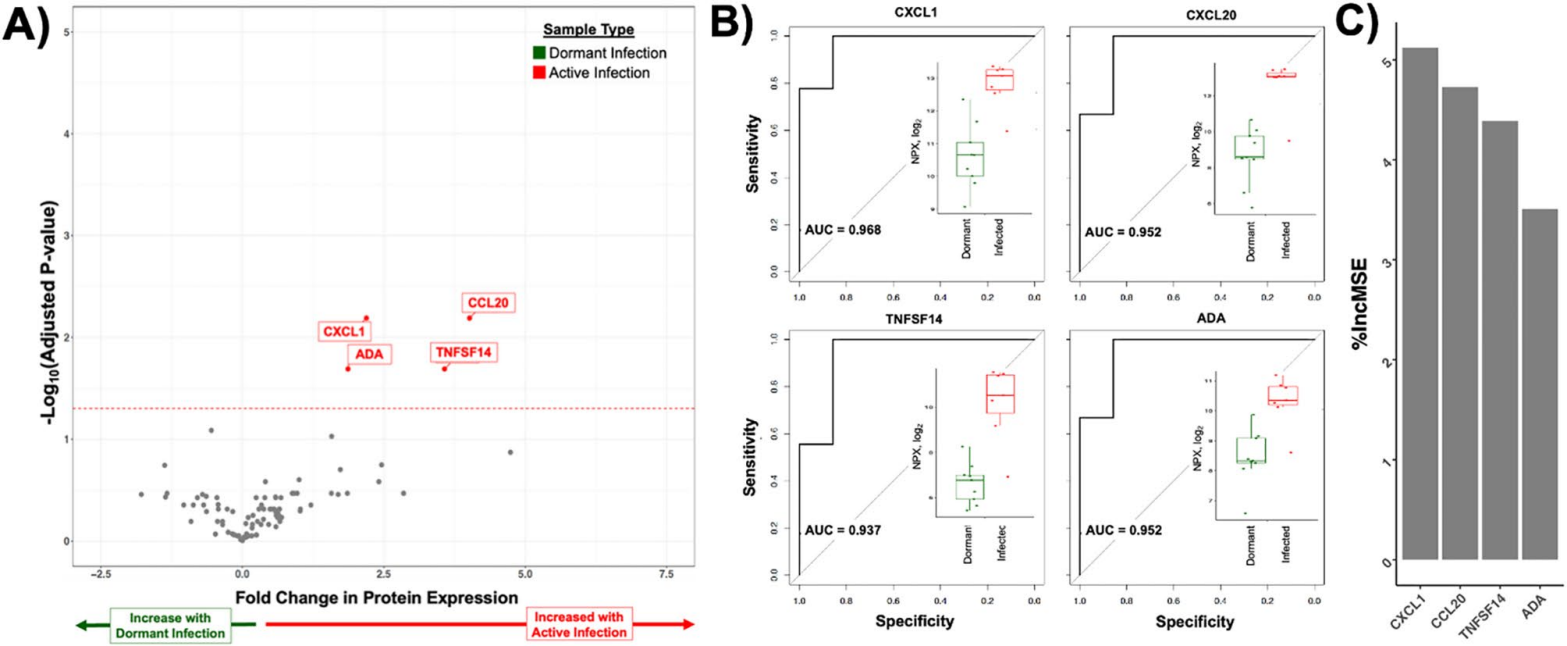
Candidate biomarkers that distinguish dormant infections from infected joint replacements. **A**) Synovial inflammatory protein expression as detected by proximity extension assay from joint replacements with a dormant infection in relationship to joint replacements with an infection demonstrating the expression of 4 candidate biomarkers for dormant infection in the synovial fluid of joint replacement patients. **B**) Accuracy as measured by area under the curve (AUC) for all candidate biomarkers. Embedded box and whisker plots showing the expression of each candidate biomarkers in normalized protein expression (NPX, log_2_) for patients with a dormant infection (green) and an infection (red). **C**) Importance of each candidate biomarker for detecting dormant infection in percentage increase in mean squared error (%IncMSE)

A complementary analysis contrasting dormant with acute infection revealed a minimal four-protein signature, including ADA, CXCL1, CCL20, and TNFSF14, each exceeding 90% accuracy and ranking among the most informative features for separating the dormant from the active state (Fig. 5, Supplemental Table 2). Again, plasma proteomes were nondiscriminatory (data not shown). Taken together, Figs. 3, 4 and 5 delineate the synovial states of infection that ranges from sterile to dormant colonization to frank intra-articular sepsis – an immunologic terrain that conventional acute phase reactant- and neutrophil-centric diagnostic criteria fail to fully detect and differentiate.

### Dormant infections reprogram the inflammatory response toward immune tolerance

**Fig. 6:**
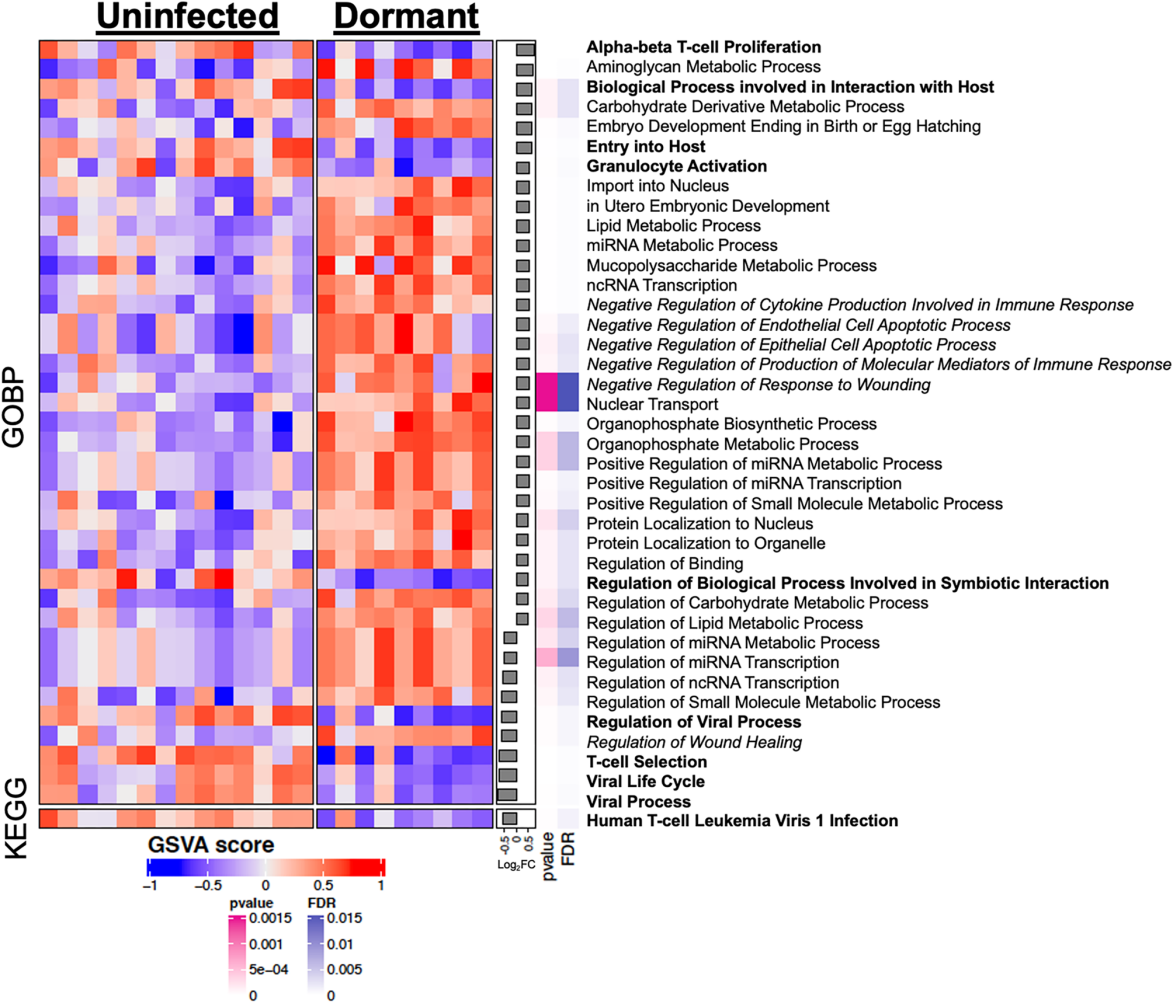
Dormant infections display signs of peripheral tolerance. Heatmap of gene set variation analysis (GSVA) using gene ontology (GO) terms and Kyoto Encyclopedia of Genes and Genomes (KEGG) pathways comparing dormant infection to joint replacements without an infection based on log fold change (Log_2_FC) as well as false discovery rate (FDR) suggesting the emergence of a distinct immune signature during a dormant infection that may involve local immune sequestration or peripheral tolerance (bolded) and wound healing, cytokine production, and negative regulation of apoptosis (italicized) within the KEGG and GO-biological process (GOBP) pathways

Pathway analysis highlighted a defense-silencing paradox within joint replacements with a dormant infection. Compared with aseptic revisions, dormant infection was characterized by programs involving viral processing, symbiotic interactions, and granulocyte activation, as well as selective down-regulation of T-cell selection and proliferation (FDR < 0.001 for both comparisons, Fig. 6, in bold). Concomitantly, programs linked to wound healing and negative regulation of endothelial apoptosis, cytokine production, and the immune response were selectively up-regulated during dormant infection (FDR < 0.001 for each comparison, Fig. 6 in italic). The net effect is an immune landscape that suggests the presence of bacteria, yet downshifts the cellular machinery needed for eradication, mirroring immune tolerance circuits described in chronic viral infections and solid tumor microenvironments.

### The absence of a dormant infection forecasts durable cure

One re-implantation originally labeled indeterminate by clustering was reevaluated against the nine-marker panel and expressed eight of nine dormant-infection proteins (89%, PDGF-B, Gal-1, CXCL11, ANGPT1, EGF, TIE2, MCP-2, and NOS3) prompting its reassignment as a dormant infection. Long-term follow-up confirmed the panel’s prognostic value over 3.0 ± 0.2 years, as two of the nine biomarker-positive joint replacements developed culture-confirmed recurrence (22%), whereas none of the fourteen biomarker-negative joint replacements (0%) relapsed over 3.2 ± 0.3 years. The panel is ideally used to discriminate among patients previously thought to be “infection free” using the 2018 MSIS criteria, which we now believe may contain both the aseptic and dormant state of infection, delivering 100% specificity and 100% positive predictive value for a future infection within three years, with a good negative predictive value (84%) and overall accuracy (70%), but lacking sensitivity (22%). Although the cohort was underpowered to identify which individual protein best predicts infection relapse, the data establish the synovial signature of dormant infection, creating a panel of proteins that are sufficient and reliable at ruling-out the presence of biofilm-embedded bacteria and are strong predictors of sustained infection-free implant survival.

## Discussion

**Fig. 7:**
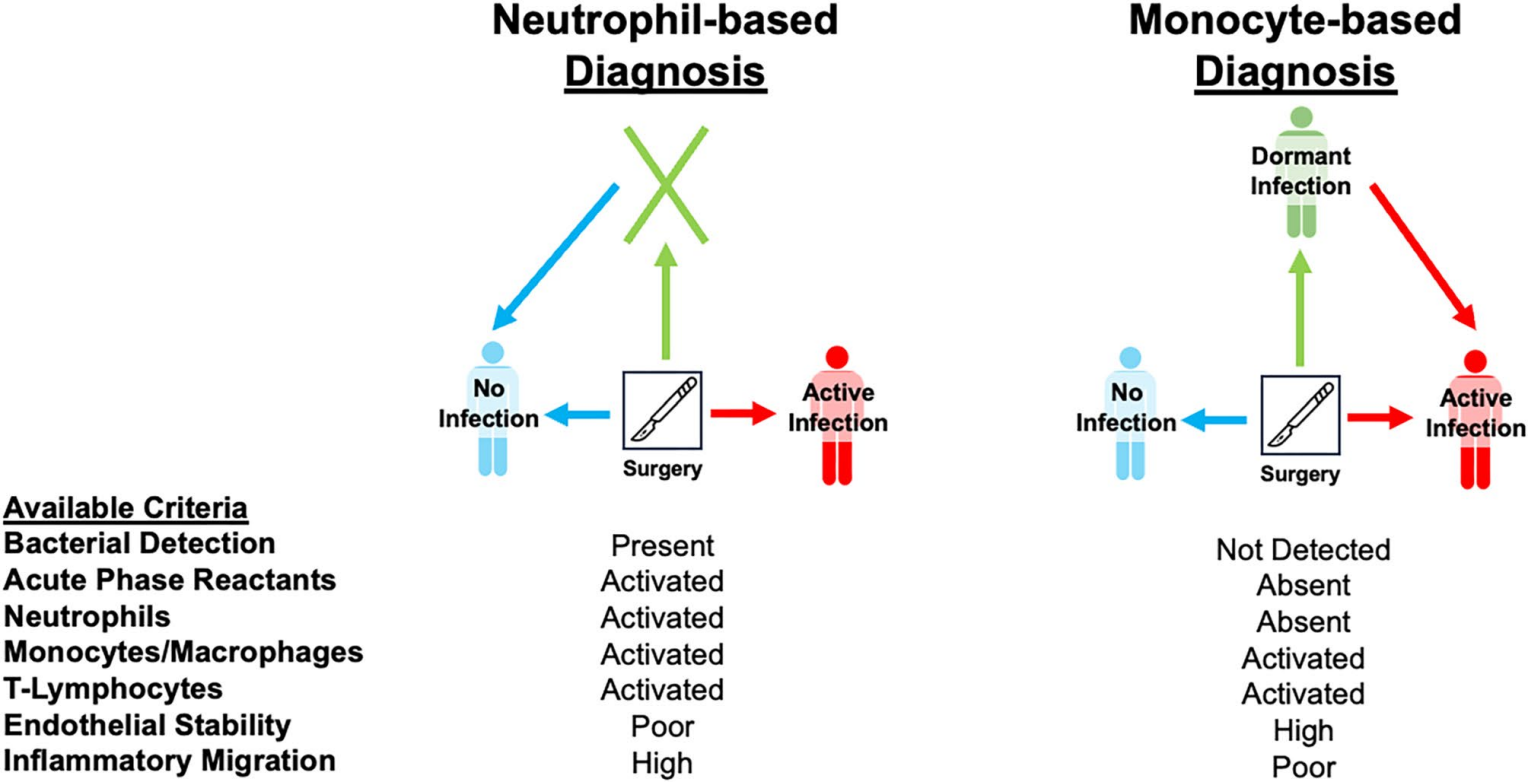
The dormant infection state is a novel paradigm of infection diagnostics. Current diagnostic tests ADDIN EN. CITE [26, 36] for infected joint replacement use the local and systemic neutrophil response to divide patients into uninfected and infection groups, but markers the mononuclear phagocyte system and endothelial stability can be used to identify joint replacements with a dormant infection that were previously characterized as uninfected using these neutrophil-based diagnostic tests. ADDIN EN.CITE [12, 26, 36] Dormant infections carried an increased risk of infection recurrence (22%) within 3 years and highlight how a pathogen can persist as an indolent reservoir for acute infection relapse and how immune profiling unmasked this silent presence. Notably, none of the dormant infections would have been flagged by neutrophil-based diagnostic criteria, ADDIN EN.CITE [26, 34–40] yet the identification of a dormant infection carried significant clinical risk of infection relapse exposing the lack of a comprehensive and sensitive panel of immunological markers that can account for the full breadth of clinically-relevant implant-associated infections when using neutrophil-based diagnostic criteria to diagnose infection

Our findings have broad implications for infection biology in general and the care of patients with implants in particular. They reveal the protein-based inflammatory signature of dormant infection in joint replacements, a state in which bacteria persist without causing overt clinical or laboratory signs of active infection (a.k.a., periprosthetic joint infection, PJI). Currently, conventional diagnostic criteria for a PJI rely heavily on acute inflammatory markers (e.g., synovial neutrophils, C-reactive protein, erythrocyte sedimentation rate) and culture-based tests detecting only the most advanced active infections that are accompanied by robust immune activation [26]. In contrast, we demonstrate that even implants deemed “infection-free” after treatment of prior infection can harbor low-grade inflammation with the distinct immunological “footprint” of lingering biofilm-resident bacteria [12, 26]. Our proteomic analysis shows that multiple inflammatory proteins remained elevated in the synovial fluid of joint replacements with a prior infection, even after treatment and MSIS screening, when compared to truly uninfected joint replacements (i.e., joint replacements with no prior incidence of infection, Fig. 2B), despite the absence of the systemic and local signs traditionally associated with an active PJI (Table 2) [26, 34–40]. The synovial profiles of the majority of joint replacements with a prior infection were largely indistinguishable from joint replacements with active infections, differing significantly in only six known inflammatory mediators. This raised the concern that many “infection-free” cases were not truly sterile, prompting us to search for the definitive inflammatory signature of dormant infection. Unsupervised hierarchical clustering confirmed the presence of a persistent inflammatory signature in 73% of “infection-free” cases that clustered with overtly infected cases (Fig. 3A), despite normal acute phase reactants (ESR, CPR) and minimal recruitment of intra-articular neutrophils, thereby defining these cases as dormant infections (Table 2) [12]. These findings are in line with our prior work using single-cell synovial transcriptomics, which suggested that 50% of joint replacements with a prior infection have dormant infections characterized by synovial CXCL5 expression mediated through activation of the IL-17 and TNF pathways [12]. In practical terms, the absence of clinical inflammation according to conventional diagnostic criteria [26, 34–40] does not guarantee the absence of infection. It may instead signal a pathogen’s success at evading, or even actively suppressing, the immune response, facilitating its survival within the host [12, 19]. This shifts the paradigm of PJI and all implant-associated infection diagnostics away from a reliance on neutrophil-driven diagnostic criteria and demonstrates that the current clinical paradigms are inadequate to describe the relevant clinical complexity of implant-associated infections (Fig. 7) [12]. Notably, none of the dormant infections identified in our cohort would have been flagged by conventional diagnostic criteria, [26, 34–40] yet the identified cases carried significant clinical risk of infection relapse, highlighting an urgent need for a more comprehensive and sensitive panel of immunological markers that can account for the full breadth of clinically-relevant implant-associated infections.

The distinct biomarker panel that characterizes the dormant infection state is a key finding of this work. We identified a set of nine synovial fluid proteins that robustly distinguishes joint replacements with a dormant infection from truly uninfected joint replacements that carry significantly less risk of recurrent infection. CXCL5 (ENA-78), a chemoattractant for neutrophils and several other types of immune cells, emerged as a hallmark of dormant infection. Immune (monocytes, macrophages, eosinophils, and neutrophils) and non-immune (endothelial, epithelial, fibroblasts, and smooth muscle) cells can all produce CXCL5. CXCL5 was similarly elevated 5-fold in active and dormant infections (12.8 ± 1.0 NPX) relative to its expression in uninfected joint replacements (10.5 ± 1.6 NPX, *p* < 0.001). The persistent overexpression of CXCL5 in the dormant state is striking, given the lack of neutrophil recruitment in joint replacements with a dormant infection, suggesting a disconnect between chemokine signaling and effector cell response. Beyond CXCL5, our machine-learning approach highlighted several other novel candidates: PDGF-B, Galectin-1, CXCL11, ANGPT1, EGF, TIE2, MCP-2, and NOS3 that each outperformed CXCL5 in discriminating a dormant infection from uninfected joint replacements (Fig. 4). Notably, two of these proteins, MCP-2 (CCL8) and CXCL11 (I-TAC), are also immune chemokines, are expressed by monocytes, T-cells, and fibroblasts, and recruit monocytes and T-cells to orchestrate tissue injury repair and modulate inflammation [48–50]. 

**Fig. 8:**
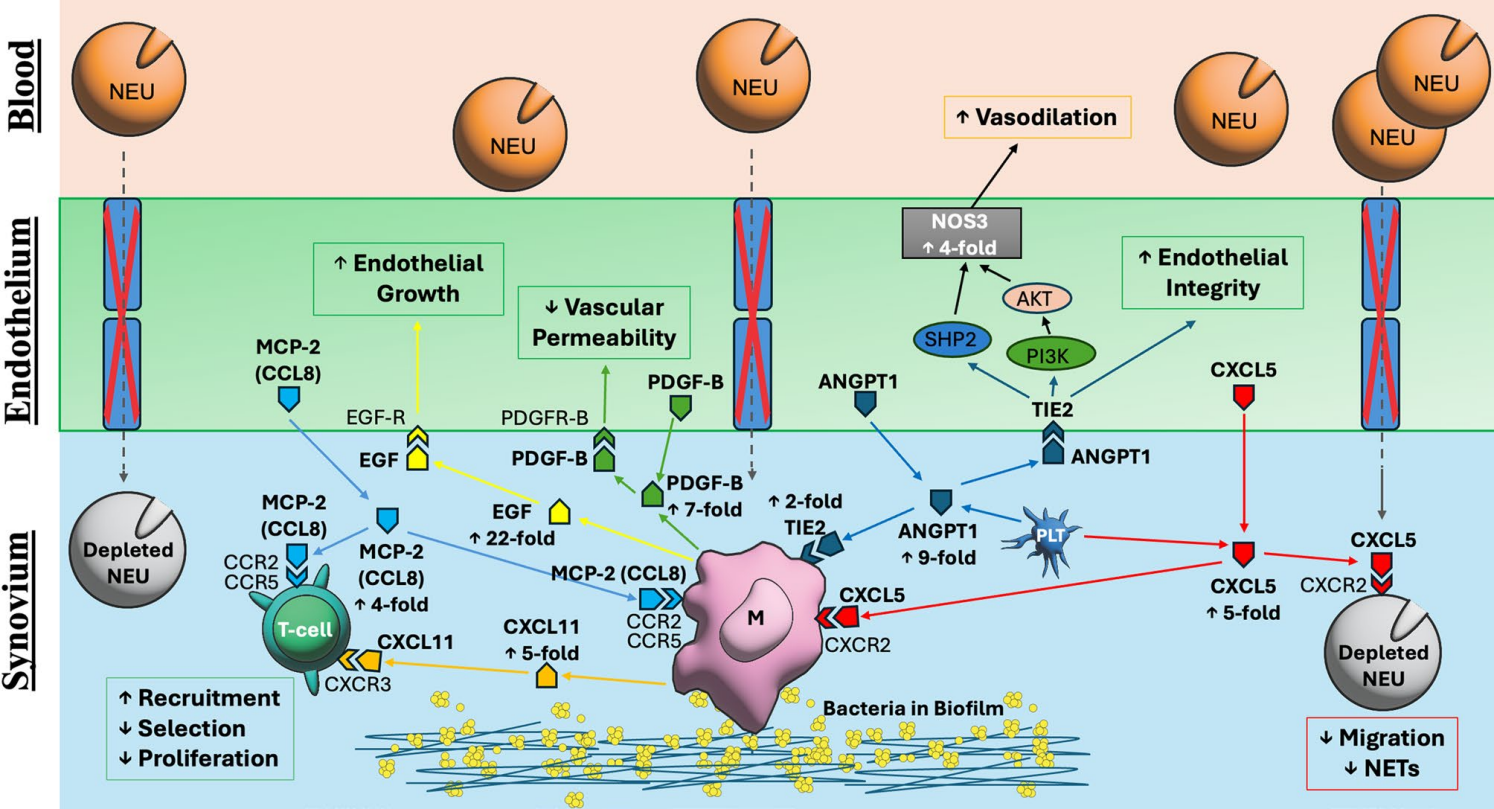
The microenvironment of a dormant infection. The chronic presence of angiogenic and immunoregulatory factors in synovial fluid may help biofilm-resident bacteria persist and shape the local microenvironment into one more permissive of bacterial survival and devoid of aggressive neutrophil-mediated destruction (NEU). To evade immune clearance, biofilm-resident bacteria interact with monocytes (M), ADDIN EN.CITE [12] vascular endothelial cells, and platelets (PLT), increasing the expression of platelet-derived growth factor (green, PDGF-B), angiopoietin-1 (dark blue, ANGPT1), tyrosine kinase with immunoglobulin and EGF homology domain 2 (dark blue, TIE2), monocyte chemotactic protein 2 (light blue, MCP-2, chemokine C-C motif ligand 8, CCL8), endothelial growth factor (yellow, EGF), chemokine CXC motif ligand 11 (orange, CXCL11), CXCL5 (red), and nitric oxide synthase 3 (black, NOS3) as secondary responses to bacterial damage. ADDIN EN.CITE [65–69] The expression of the angiogenic and immunoregulatory factors (detected enzymes and cytokines in the labeled in the biomarker panel for dormant infection are bolded with measured fold change, while receptors and enzymes not in bold indicate in the biomarker panel for dormant infection are marked using regular text) within the synovial fluid serve to stabilize the vascular endothelium and prevent cellular migration from the bloodstream to the synovial space over time. This local sequestration (red X and dotted line at junction between endothelial cells) during a time of local neutrophil (grey) depletion and loss of neutrophil extracellular trap (NET) formation ADDIN EN.CITE [12] as a result of ongoing planktonic bacterial control helps explain the increased synovial CXCL5, CXCL11, and MCP-2/CCL8 levels, lack of systemic acute phase reactant expression, and continued monocyte and T-cell activity previously shown to be indicative of dormant infection. ADDIN EN.CITE [12]

In deciphering, “why dormant infections remain clinically silent,” our data point to mechanisms of peripheral immune tolerance. Joint replacements with a dormant infection showed a downregulation of pathways related to neutrophil activation and T-cell proliferation compared to uninfected joint replacements. One might expect an infection to provoke inflammation; instead, dormant infection exhibited a subdued immune transcriptomic profile, as if the immune system had been actively dampened [12]. Gene set analysis confirmed a distinct immunologic signature consistent with peripheral immune tolerance in the local environment (Fig. 6). Notably, Galectin-1 (Gal-1) normally promotes immune tolerance and limits inflammation, so the downregulation of Gal-1 in joint replacements with a dormant infection suggests an ongoing antibacterial response [51]. Hence, in the context of dormant infection, a chemokine-driven feedback loop sustains a chronic inflammatory state that neither eradicates the bacteria nor allows full inflammatory resolution. This equilibrium, an immune “truce” between host and microbe, mirrors phenomena observed in chronic viral infections, cancer, and commensal colonization where the host and pathogen coexist in a state of muted inflammatory conflict [52–57]. Notably, the concept of a dormant infection has parallels beyond orthopaedic surgery and PJI. Diseases like tuberculosis feature latent infections where pathogens persist in a granuloma with the host immune system in an immunologic stalemate (Fig. 8) [58, 59]. Likewise, staphylococcal infections in other implanted devices may have analogous dormant phases [60, 61]. Thus, our findings are broadly relevant to infection biology and immunology, illustrating how a pathogen can persist as an indolent reservoir for acute infection relapse and how immune profiling unmasks this silent presence.

We speculate that biofilm-resident bacteria in a persistent state actively modulate the synovial immune milieu to avoid terminal clearance, much as cancer stem cells create an immunosuppressive microenvironment to evade immune clearance. The parallel between dormant infection persistence and cancer stem cell immunity is compelling. For example, increased expression of CXCL5 in the tumor microenvironment has been linked to activation of the CXCR2 pathway and recruitment of pro-tumor immune cells, thereby promoting tumorigenesis and angiogenesis, demonstrating that CXCL5 expression drives glioblastoma progression [62]. Elevated CXCL5 is associated with poor patient survival, underscoring how a sustained chemokine signal can facilitate pathological persistence [63, 64]. In our study, the chronic presence of CXCL5 (and related angiogenic/immunoregulatory factors) in synovial fluid may similarly help bacteria persist and shape the tissue microenvironment into one more permissive to bacterial survival and devoid of aggressive inflammatory destruction. The expression of platelet-derived growth factor (PDGF-B), angiopoietin-1 (ANGPT1), TIE2 (ANGPT1 receptor, tyrosine kinase with immunoglobulin and EGF homology domain 2, endothelial tyrosine kinase, TEK), endothelial growth factor (EGF), and nitric oxide synthase 3 (NOS3) from endothelial cells and fibroblasts can be triggered as secondary responses to bacterial damage, [65–69] but also serve to stabilize the vascular endothelium and prevent cellular migration from the bloodstream to the synovial space over time. Vascular sequestration during a time of local neutrophil depletion helps explain the increased CXCL5, CXCL11, and MCP-2 levels, lack of systemic acute phase reactant expression, and continued monocyte and T-cell activity previously shown to be indicative of dormant infection (Fig. 8) [12]. 

When combined as a panel, these 9 biomarkers provide high sensitivity and carry a high positive predictive value for forecasting infection relapse within three years in a patient previously thought to be “infection free” using the 2018 MSIS criteria. In practical terms, if none of these markers were elevated in a patient’s synovial fluid, we observed no infection recurrence within 3 years. Conversely, elevation of the dormant infection markers, alongside reduction of Galectin-1, carried an increased risk of infection recurrence (22%) within 3 years. Because not every biomarker-positive case progressed to clinical infection in the observed timeframe, the panel’s sensitivity and negative predictive value was moderate, technically indicating the potential for some “false negatives,” or possibly indicating that many more cases remain dormant with quiescent microbes that continue to remain immunologically contained until future relapse beyond the observed timeframe. Nonetheless, the high specificity of this panel makes it a powerful rule-out test that can, with high confidence, identify patients truly free of infection, which is crucial for safe re-implantation surgery. Equally important, it provides a novel, culture-independent means of detecting an occult infection that traditional microbiological methods or inflammatory markers would miss [26, 34–40, 70, 71]. 

These insights carry four critical clinical implications. First, diagnosing a dormant infection could become a reality using this biomarker approach [12]. Second, a biomarker-positive result would alert clinicians to residual infection risk even if traditional markers are negative (e.g., synovial neutrophils, C-reactive protein, erythrocyte sedimentation rate) and result in more prompt and more aggressive management, for example by extending the course of antibiotics, revising surgical strategy to ensure more complete biofilm removal, or delaying re-implantation to give additional time for more durable infection control [72, 73]. Third, a biomarker-negative result provides strong confidence that the joint is truly infection-free, supporting safe definitive re-implantation [24]. Indeed, in our cohort, patients without the synovial immune signature of dormant infection had 0% infection relapse within the observed 3-year timespan. Fourth, in terms of prognosis, identifying a dormant infection might warrant closer postoperative surveillance, or even prophylactic monitoring, to catch relapse early, as is the case with current cancer management strategies. The ability to predict which patients are at high risk of relapse moves us closer to precision medicine for implant-associated infections.

Beyond diagnostics, recognizing the roles of peripheral immune tolerance and vascular sequestration in establishing implant-associated dormant infection suggests multiple novel therapeutic avenues. If bacteria can persist by inducing local immune suppression, then intentionally modulating the immune response could help eradicate them as well. This concept is analogous to cancer immunotherapy, where the goal is to break the immune evasion in the tumor microenvironment. One could imagine adjunct treatments that disrupt the tolerant niche or boost local immunity. For example, therapies that disrupt the CXCL5-CXCR2 (SB225002, AZD-8309) [74, 75] or ANGPT1/TIE2 (BowANG1, AKB-9778) [76, 77] axes are already under exploration and might be repurposed to prevent bacteria from exploiting the dormant state. Alternatively, localized immune stimulation at the affected joint could be considered. There is also interest in whether immune checkpoint pathways, such as the programmed death-ligand 1 (PD-L1) pathway, may play a role in bacterial persistence; if so, checkpoint inhibitors or other immunomodulators could theoretically be employed to tip the balance in favor of the host immune system.

This study has limitations that should be considered when interpreting the results. First, we conducted this pilot study under the presumption that biofilm that may not be easily found by clinically available culture methods due to the slow growth rate and auxotrophic culture conditions of biofilm-resident bacteria and the possibility of sampling bias at the time of surgery, so we did not seek to provide direct evidence of local biofilm per se, such as microscopic analysis of spacers, and instead chose to create a novel set of immunologic biomarkers that would bypass these microbiologic deficiencies and increase the clinical utility of our new paradigm. Second, this is a pilot study, and the corresponding sample size is relatively small, with only 30 analyzed patients. This limited sample size can introduce sampling bias, affecting the generalizability of the findings. Larger, more diverse cohorts are needed to validate the results and ensure they are representative of the broader population after multivariate control. The actual number of samples required to validate our findings in diagnostic testing depends on the AUC of the test and the prevalence of the detected disease. We estimate 20–30 samples would be required to validate each candidate biomarker for dormant infection at a rate of 10% and 50–100 samples for active infection at rate of 2%. Third, while the molecular methods presented here offer a culture-independent approach to detecting infections, they cannot identify which bacterial species are present. Due to the slow-growing and difficult-to-culture nature of biofilm-resident bacteria, culture-based methods of identification are also problematic, and as such bacterial DNA/RNA sequencing of the synovial fluid would be better suited to characterize the microbial landscape. This limitation underscores the need for complementary methods to provide a comprehensive understanding of infection dynamics. Fourth, the Olink Immuno-Oncology Panel is cytokine-heavy and does not capture matrix-degrading enzymes, which may also serve as biomarkers of dormant infection that we did not directly test. Fifth, we did not account for variations in pre- and postoperative prophylactic treatments and other medical interventions, which could modulate the inflammatory response and influence the study outcomes. Standardizing these factors or adjusting for them in the analysis is crucial for more accurate interpretations. Sixth, we identified associations between the proteomic profile and dormant infection, but these associations do not establish causality. Future studies should focus on validating our findings with a larger cohort and longitudinal study to assess the prevalence, characteristics, and progression of dormant infections in joint replacements, as well as the possibility of temporal resolution of dormant infections. Multicenter studies involving diverse patient populations and adding bacteria-specific signatures, will help establish the sensitivity and specificity of the proposed biomarkers with respect to more traditional diagnostic tests.

In summary, this study unmasks a highly prevalent, yet previously invisible immunologic footprint of dormant infection, providing proof-of-concept that the host immune response can be leveraged to detect the presence of an occult, persistent, likely immunosuppressive microenvironment used by bacteria to evade immune clearance, predicting an increased likelihood of acute infection recurrence [12, 19–22]. These findings challenge the use of conventional clinical diagnostic criteria, which are over-reliant on acute phase reactants and neutrophil recruitment, and creates a new prognostic clinical paradigm that now includes precision, immune-guided management of dormant infections likely present in many types of implant-associated infections, like caterers, ports, meshes and pacemakers. It is estimated that 3.6 million joint replacements were performed worldwide in 2023 [78, 79]. The reported incidence of an active infection after a joint replacement ranges from 1 to 2%, meaning there are as many as 72,000 new active infections generated annually [80]. Based on our results, active infections emerge from dormant infections and represent only 22% of the global incidence of dormant infections, so we predict over 327,272 new dormant infections annually, or, stated more bluntly, roughly 10% of all joint replacements were performed worldwide harbor a dormant infection [12]. Assuming synovial testing for the biomarkers of dormant infection is implemented only when the 72,000 new active infections are subsequently “infection-free” (according to the 2018 MSIS-criteria) and ready for re-implantation, [26] we would then estimate detecting an additional 59,000 residual dormant infections prior to reimplantation (82% − 9 dormant infections detected from 11 MSIS-cleared joint replacements) and possibly prevent 13,000 to 16,000 relapses (18% − 2 infection relapses from MSIS-cleared joint to 22% − 2 infection relapses from 9 dormant infections) saving $152 to $187 million in re-infection related treatment costs [81, 82]. Of course, we also hope that synovial testing for the biomarkers of dormant infection can be achieved for less than other commercially available non-prognostic tests that only diagnose active infection, like Synovasure ($200 to $850, Zimmer-Biomet, Warsaw, IN), Joint-ID ($100 to $600, Vikor Scientific, Charleston, SC) or OrthoKey ($300, MicroGen Diagnostics, Orlando, FL). We envision five future directions to build on this work. First, retrospective larger series are needed to validate the performance of the biomarker panel and determine its positive predictive value in a broader population of implant-associated infections. Second, refining the panel to a single practical test will be important for widespread clinical adoption. Third, mechanistic studies are warranted to understand how these proteins contribute to immune evasion. Fourth, time-course studies should examine how dormant infections evolve over time to inform optimal timing for intervention. Fifth, immunomodulatory therapies should be explored in randomized controlled trials to determine safety and efficacy in this patient population. Ultimately, diagnosing and clinically addressing dormant infection before relapse would significantly improve the success of long-term implant retention and infection-free patient outcomes, turning what was once an unseen threat into the next target for precision infection management.

## Supplementary Information

Below is the link to the electronic supplementary material.

Supplementary Material 1

Supplementary Material 2

## Data Availability

The datasets generated and analyzed during this study are not publicly available since this was collected under institutional review board approval that preserves patient anonymity and protect against blanket disclosure. As a result, the datasets generated and analyzed during this study are available from the corresponding author only after reasonable request and implementation of the appropriate safeguards to protect patient anonymity.
